# Time-heterogeneity of the Förster Radius from Dipole Orientational Dynamics Explains Observed Dynamic Shift

**Published:** 2024-04-15

**Authors:** David Frost, Keisha Cook, Hugo Sanabria

**Affiliations:** School of Mathematical and Statistical Sciences, Clemson University.; School of Mathematical and Statistical Sciences, Clemson University.; Department of Physics and Astronomy, Clemson University.

## Abstract

Förster resonance energy transfer (FRET) is a quantum mechanical phenomenon involving the non-radiative transfer of energy between coupled electric dipoles. Due to the strong dependence of FRET on the distance between the dipoles, it is frequently used as a “molecular ruler” in biology, chemistry, and physics. This is done by placing dipolar molecules called dyes on molecules of interest. In time-resolved confocal single-molecule FRET (smFRET) experiments, the joint distribution of the FRET efficiency and the donor fluorescence lifetime can reveal underlying molecular conformational dynamics via deviation from their theoretical Förster relationship. This deviation is referred to as a dynamic shift. Quantifying the dynamic shift caused by the motion of the fluorescent dyes is essential to decoupling the dynamics of the studied molecules and the dyes. We develop novel Langevin models for the dye linker dynamics, including rotational dynamics, based on first physics principles and proper dye linker chemistry to match accessible volumes predicted by molecular dynamics simulations. By simulating the dyes’ stochastic translational and rotational dynamics, we show that the observed dynamic shift can largely be attributed to the mutual orientational dynamics of the electric dipole moments associated with the dyes, not their accessible volume.

## I. | INTRODUCTION

Förster resonance energy transfer (FRET)[1, 2] experiments are commonly used in biochemistry and biophysics to measure distances on molecules of interest.[3–6] This is done by exploiting the physical mechanism of FRET wherein energy is transferred non-radiatively between a *donor* and an *acceptor* fluorophore due to electric dipole coupling. Throughout the document, these fluorophores will be referred to as dyes. The rate of energy transfer depends on the distance between the dipoles to the sixth power, as well as an orientational factor and several other time-independent factors.[1, 4, 7–9] Due to this strong dependence on distance, FRET is often used as a *molecular ruler* [10, 11]. Therefore, an accurate understanding of FRET measurements is paramount to understanding the collected experimental data and forming concrete hypotheses about the conformational dynamics of the molecule of interest, which provides information for the health sciences. [12]

It has been shown in [13–15] that the joint distribution between FRET efficiency and lifetime measurements contains a wealth of information on the structural dynamics of the underlying molecule. This information is seen by a deviation from the ideal Förster relationship, termed a dynamic shift [13]. The use of smFRET in structural biology is well established. [11, 14, 16–33] Moreover, the use of the dynamic shift to understand dynamic structural biology has recently been established. [14, 21, 30, 31, 34, 35] Due to thermal noise, the dye position will fluctuate randomly, inducing a change in the joint FRET-lifetime distribution.[15, 36] Hence, it is necessary to understand the influence of this stochastic fluctuation on the resulting FRET measurements.[37–41] So, a complete understanding of the dynamic shift induced by the dye dynamics is needed to facilitate accurate estimates for structural dynamics.[42, 43]

Currently, in the literature, there is no physical model for the dye motion to facilitate accurate and high-precision FRET measurements.[15, 36] The models available are either simplistic [44] or require a high computational cost [20, 43, 45, 46]. Typically, all-atom molecular dynamics simulations are conducted on a sample-by-sample basis to calculate the accessible volume of the dyes in order to estimate the range of motion of the molecule and, therefore, provide a way of uncertainty quantification in the FRET-lifetime distribution [31, 34, 43, 47]. An important critique of this method is that the simulations do not last for the entire sampling process.[43] Hence, the full range of motion for the dyes during a sample time is not achieved. Here, we attempt to provide a semi-analytic model for fluorescent dye motion. In addition, these simulations are meant to approximate the equilibrium distribution of the dye motion and hence ignore any time inhomogeneity in the energy transfer process itself. We consider several of the following relevant aspects of dye motion. First, will an isotropic Gaussian process provide a correct model? In this situation, the common assumption of Gaussian inter-dye displacement is satisfied. Second, what is the impact of linker length? Third, what is the role of dipole motion in FRET measurements? We answer these questions in the context of a simulated smFRET experiment. Our results shed light on the past assumption that the dynamic shift induced by the dyes is due to the accessible volume of the dye. [13, 14] In reality, the dynamic shift has to do with the full state dynamics of the dyes, both translational and orientational.

### A. Confocal smFRET

A time-resolved confocal smFRET experiment uses the FRET phenomena by attaching fluorescent dyes to a molecule of interest by continuously exciting the donor molecule and measuring the resulting emitted light; one may estimate the FRET efficiency and, from that, estimate the distance between the molecules.

The estimation of FRET efficiency is done in two ways deemed *intensity* based FRET and *lifetime* based FRET.[4, 48] Both methods may be used in time-resolved confocal smFRET, and indeed, the subject of this paper concerns the relationship between the two.

The experiment is carried out by diluting the sample of interest so that, on average, less than one molecule of interest lies within the confocal volume of the microscope.[4, 48] By freely diffusing through the confocal volume, the attached donor dye becomes repeatedly excited, and the resulting fluorescence is measured. The random amount of time spent within the confocal volume is termed a burst time due to the burst of photons seen in the measurement process. Moreover, by recording the time between the laser pulse and measurement, one obtains the *lifetime* measurement [48]. The lifetime measures how long the molecule remains excited, a quantity that will change in the presence of other molecules.

A *burst time* is defined as the amount of time the molecule diffuses through the confocal volume. During this time, the inter-photon arrival time decreases sharply from the background level. Through this stochastic time frame, measurements are made and one obtains a sample of lifetimes and photons by which the FRET efficiency and lifetime distribution may be estimated. By collecting multiple burst samples, one obtains the joint FRET-lifetime distribution. During each burst, the donor molecule repeatedly enters an excited state wherein it may fluoresce, transfer energy via the FRET mechanism, or relax due to some other relaxation pathway. We refer to the event of excitation and the following process dictating the observation as an *excitation event*. In simple terms, a burst time will give a data point in the FRET-lifetime distribution and represents *multiple* photons. Whereas an excitation event gives the color and lifetime of *one* photon.

### B. FRET Model as a Continuous-Time Markov Chain (CTMC)

Consider two completely static dyes with normalized dipole moments $\mu_{A}\in S^{2}$ and $\mu_{D}\in S^{2}$ for the acceptor and donor, respectively. Further, let the inter-dye displacement vector be $r\in R^{3}$. The energy transfer rate is defined in Eq. (1),

(1) $$k_{ET}\left(r\right)=k_{D}{\left(\frac{R_{0}}{∥r∥}\right)}^{6},$$

where $k_{D}$ is the fluorescence decay time for the donor dye, and $R_{0}$ is a physical parameter called the Förster radius where the probability of energy transfer is 0.5 [2, 4, 17, 41]. Note that the energy transfer rate increases dramatically as the distance decreases and vice versa as the distance increases. However, no matter the distance, no energy transfer can happen if $\mu_{D}$, $\mu_{A}$, and $\overset{ˆ}{r}$ are mutually orthogonal. [49, 50] The Förster radius can be written as $R_{0}^{6}(t)=C\kappa^{2}(t)$ where C is a constant depending on the environment surrounding the dye. The parameter $\kappa^{2}(t)$ is the dipole orientational factor

(2) $$\kappa (t)=\left(\mu_{D}(t)\cdot \mu_{A}(t)\right)-3\left(\overset{ˆ}{r}\cdot \mu_{D}(t)\right)\left(\overset{ˆ}{r}\cdot \mu_{A}(t)\right)$$

with $\overset{ˆ}{r}=\frac{r}{∥r∥}$.[51]. Since the dipole moments are known to reorient on timescales faster than the energy exchange rate[50, 52], kappa square is treated as time-dependent. This is in contrast to previous models wherein the dipole moment is chosen from the equilibrium distribution of the rotational diffusion. [41, 53, 54] In this model, the initial distribution of the dipoles is chosen according to the equilibrium distribution, but rotational processes evolve during the energy transfer. This is vital because the FRET efficiency cannot be evaluated in terms of an evaluation of the energy transfer rate at a specific time but rather as dependent on the history of the $\kappa^{2}$ process using the fact that the transfer times at a time T>0 of a nonhomogeneous CTMC are exponential with rate $\int_{0}^{T}k(s)ds$ [55–58] therefore the probability a donor photon is fluoresced before energy transfer, termed the FRET efficiency, at time T is given by

(3) $$ℰ\left(T\right)=\frac{\int_{0}^{T}k_{ET}\left(s\right)ds}{\int_{0}^{T}k_{ET}\left(s\right)ds+k_{D}T}.$$

In this way, the FRET efficiency process, ℰ(t), is non-Markovian. It is important to note that each excitation event’s fluorescence process will still be Markovian. As noted in [53], the inter-arrival time for the photon count process need not be exponentially distributed.[59] Therefore, the photon arrival process cannot be seen as a time-homogeneous Poisson process in contrast to previous common assumptions.[3, 23, 60–62] Depending on the rate at which the dyes reorient, each vector may be treated as uniformly distributed on the unit sphere or a cone.[53] In this case the average value of $\kappa^{2}$ is given by $\frac{2}{3}$.[1] This is referred to as the dynamic averaging regime [50, 51, 53].

**FIG. 1:**
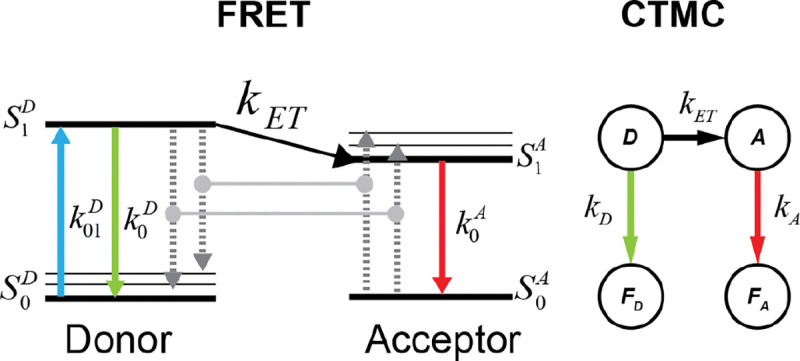
Representation of FRET by CTMC. Comparison of the CTMC states with the Jablonski diagram for the FRET process.

Using the fact that exponential random variables can well model fluorescence times [63] and accounting for the time dependence of the Förster radius on $\kappa^{2}$ the energy transfer process in FRET is modeled as a time-inhomogeneous continuous-time Markov chain (CTMC)[56, 57], illustrated in Figure 1, with rate matrix defined in (4);

(4) $$Q(t)=\left(\begin{array}{cccc} -\left(k_{D}+k_{ET}(t)\right) & k_{ET}(t) & k_{D} & 0\\ 0 & -k_{A} & 0 & k_{A}\\ 0 & 0 & 0 & 0\\ 0 & 0 & 0 & 0 \end{array}\right),$$

where $k_{D}$ is the Donor fluorescence rate, $k_{A}$ is the acceptor fluorescence rate, $k_{ET}$ is the FRET energy transfer rate. The state space is defined as $S=\left\{D,A,F_{D},F_{A}\right\}$, where D is the donor position, A is the acceptor position, $F_{D}$ is the donor fluorescence, and $F_{A}$ is the acceptor fluorescence.

Note that if r=0, the CTMC is reduced to a two-state system transitioning between states A and $F_{A}$ with rate $k_{A}$.

Assuming the dynamic averaging regime, one may easily derive the common time-homogeneous FRET efficiency. Observation of an acceptor photon only occurs when energy transfer occurs, i.e., if we have a transition from D→A. Let $\tau_{D}$ and $\tau_{ET}$ be the transfer times of $D\to F_{D}$ and D→A respectively. Then using Equation 3 one obtains,

$$ℰ(t)\;=P\left(min\left(\tau_{D},\tau_{ET}\right)=\tau_{ET}\right)\;\;=\frac{\int_{0}^{t}k_{ET}(t)}{\int_{0}^{t}k_{ET}(t)+k_{D}t}\;\;=\frac{k_{D}\left(\frac{R_{0}}{r}\right)t}{k_{D}\left(\frac{R_{0}}{r}\right)t+k_{D}t}\;\;=\frac{1}{{\left(\frac{r}{R_{0}}\right)}^{6}+1}.$$

Therefore, the theoretical time-homogeneous FRET efficiency is given by

(5) $$ℰ=\frac{1}{{\left(\frac{r}{R_{0}}\right)}^{6}+1}.$$

One may approximate this value using two methods: intensity-based FRET and lifetime-based FRET [41, 48]. For *intensity-based FRET*, the measurements are counts of observed photons from each dye. Effectively, the experiment measures the probability of success of a binomial random variable with a probability of success p given by the FRET efficiency, ℰ. The best estimator in the absence of experimental corrections is given by the number of successes observed divided by the total number of trials, denoted in Equation (6);[31, 41, 64–66]

(6) $${ℰ}_{I}=\frac{I_{A}}{I_{A}+I_{D}}.$$

For *lifetime-based FRET*, consider

$$ℰ+P\left(min\left(\tau_{D},\tau_{ET}\right)=\tau_{D}\right)=1.$$

Noting that since $P\left(\tau_{D}>t|min\left(\tau_{D},\tau_{ET}\right)=\tau_{D}\right)∼exp\left(k_{D}+k_{ET}\right)$ [57], the FRET efficiency can be calculated in terms of the lifetimes,

(7) $$ℰ=1-\frac{\tau_{D}^{\prime }}{\tau_{D}},$$

where $\tau_{D}^{\prime }={\left(k_{D}+k_{ET}\right)}^{-1}$ is the lifetime of the donor in the presence of the acceptor, and $\tau_{D}=k_{D}^{-1}$ is the lifetime in the absence of the acceptor. Hence, the measurements are observed lifetimes and an estimate for the mean lifetime of the donor, $\tau_{D}$. The FRET efficiency is estimated by approximating the mean, and hence the rate, of this exponential random variable [48].

### C. The Dynamic Shift

Consider a sample drawn from a population with a distribution of fluorescence rates K(x) such that the probability of an individual having a specific rate is given by the distribution π(x). Then the average lifetime is

(8) $$\overset{‾}{\tau }=E\left[\tau \right]=\int_{R^{d}}E\left[\tau |K\left(x\right)\right]d\pi \left(x\right)=\int_{R^{d}}\frac{1}{K\left(x\right)}d\pi \left(x\right).$$

However, the lifetime resulting from the average rate is given by

(9) $$\underline{\tau }=\frac{1}{E[K(x)]}=\frac{1}{\int_{R^{d}}\;K(x)d\pi (x)}.$$

Therefore, by Jensen’s inequality [67], using the fact that $\phi (x)=\frac{1}{x}$ is convex for x∈[0,∞), it must be that

(10) $$\underline{\tau }=\frac{1}{E[K(x)]}\leq E\left[\frac{1}{K\left(x\right)}\right]=\overset{‾}{\tau }.$$

Consequently, the average lifetime for a mixture of states will be greater than that of the associated average state. This phenomenon is known as the dynamic shift.

**FIG. 2:**
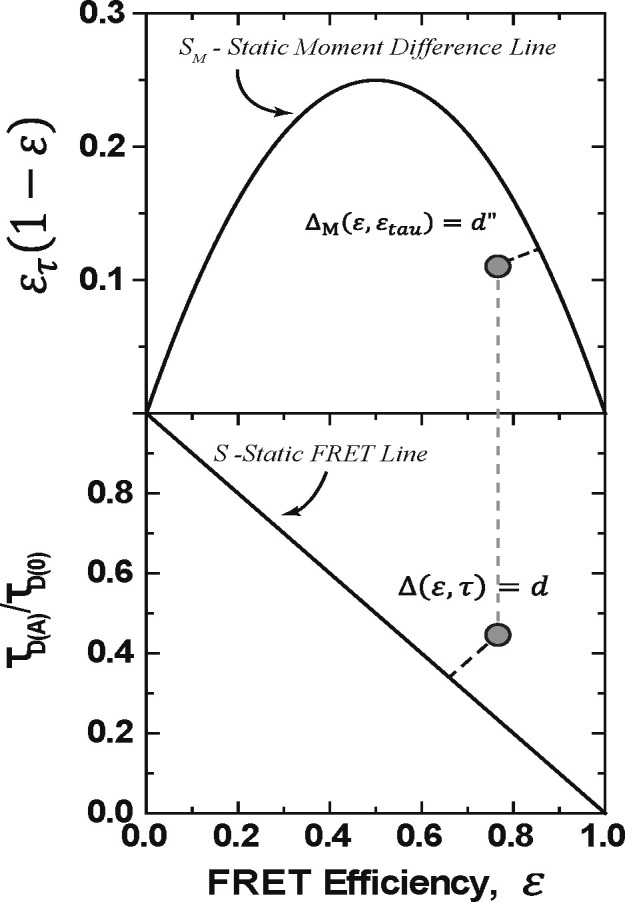
Visualization of the definition of the dynamic shift using normalized values: $S=\left\{(ℰ,\tau )\in (0,1{)}^{2}:ℰ+\right.\;\tau -1=0\}$ in the bottom. The top figure is a visualization of the moment difference dynamic shift. For the moment difference We used ${ℰ}_{\tau }=1-\frac{\overset{-}{\tau_{D(A)}}}{\tau_{D(0)}}</Figure\_Caption>$

We introduce a new quantitative definition of the dynamic shift Δ for a point $\left({ℰ}^{\prime },\tau^{\prime }\right)$ in the plane, given by the signed distance from the point to the static line, S={(ℰ,τ):ℰ=1-τ} as shown in Figure 2.

One can find an expression for the dynamic shift using standard analytic geometry. The formula in the normalized lifetime case, $\frac{\tau_{D(A)}}{\tau_{D(0)}}=\tau$, is given by

(11) $$\Delta \left(ℰ,\tau \right)=\frac{ℰ+\tau -1}{\sqrt{{ℰ}^{2}+\tau^{2}}}.$$

The definition of the dynamic shift becomes the signed length of the orthogonal projection of the point onto the static line - how much it deviates from the static line. Under the constraint that the FRET - lifetime pair resides within the unit square, this implies that the dynamic shift has extreme values at $\pm \frac{1}{\sqrt{2}}$ at (1, 1) and (0, 0). This definition provides a means by which each data point from a smFRET experiment may be assigned a dynamic shift value, and the resulting distribution may be examined. The average dynamic shift can be seen as an average deviation from the static line. With two state transitions, this definition agrees with the definition present in [13]. Furthermore, when the average dynamic shift is 0, one may use the dynamic shift distribution to quantify shot noise inherent in the measurements.

Another way to view the dynamic shift introduced in [13] is the moment difference approach. In this method, one investigates the behavior of the difference between the first and second moments of the FRET distribution, E[ℰ(1-ℰ])=E[τ(1-τ)]. In this way, the effects of multiple states are linearized, while the static line is nonlinear. In this case, the dynamic shift can be seen as a consequence of Jensen’s inequality but for concave functions. When dynamic mixing is present in the sample, the moment difference should fall below the static line of ${ℰ}_{\tau }(1-ℰ)$. Note that when this difference is negative, it implies that the covariance between ${ℰ}_{\tau }$ and ℰ is larger than the average of ℰ. This can occur from shot noise or when the lifetime distribution has a large variance but maintains the same mean. Conditions for this to occur are discussed in Section IIIB To define the dynamic shift from the static moment difference line, one again takes the distance from the point to the static line. The vector between the point and the static line with a length equal to the moment difference dynamic shift will be orthogonal to the tangent line of the static line at the point closest to the point.

The dynamic shift introduced in [13] considers an underlying distribution dependent on two separate states. Consider two FRET efficiency states denoted by ${ℰ}_{i}$, i=1,2 with equal transition rate between the states λ for simplicity. Such a two-state system provides valuable insight into the nature of the dynamic shift. When two states are separated on long time scales, λ<<1, the dynamic shift is slight due to the small amount of mixing during a burst or sample. As the two states mix, corresponding to an increase in λ, an arc forms between the static FRET-lifetime coordinates, following $\left(1-{ℰ}_{1}-{ℰ}_{2}\right)ℰ-{ℰ}_{1}{ℰ}_{2}$. As λ→∞, this process culminates in a point mass FRET-lifetime distribution with a dynamic shift at the maximum of this arc. Therefore, the dynamic shift can be seen as a metric of the amount of mixing between states. Two-state transition systems can be used to understand the transition rates between stable states in protein conformational dynamics. For the current purpose, it provides a convenient method for interpreting the dynamic shift induced by the dyes. The dynamic shift will most readily be present when there are mixing states, and understanding the influence of mixing between an uncountable number of states is of current interest. It will be shown in Section III that the dynamic shift induced by dye dynamics can be viewed as a consequence of the fluctuations in the energy transfer rate during the FRET process. Under common circumstances, the energy transfer rate can be approximated by a two-state system corresponding to the modes of the distribution, essentially leading to a quickly transitioning two-state system.

## II. STOCHASTIC MODELS OF FLUORESCENCE DYNAMICS

**FIG. 3:**
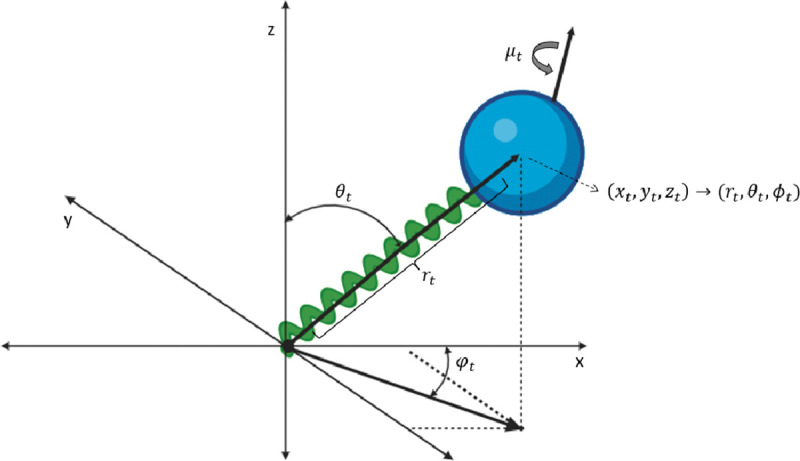
Cartoon showing the coordinate references for the processes. The translational process is expressed in both Cartesian and polar coordinates with $\phi_{t}$ the azimuthal coordinate, $\theta_{t}$ the polar coordinate, $r_{t}$ the radial coordinate, $\left(x_{t},y_{t},z_{t}\right)$ standard Cartesian coordinates, $\mu_{t}$ the dipole orientational process

This section presents several models of stochastic fluorescence dynamics related to the smFRET dynamic shift and associated molecular probes. In this way, estimation of the mixture of states, π(x) as seen in Section IC, is accomplished. Figure 3 shows the basic coordinate expression for the dye motion.

Throughout, the dynamics are assumed to evolve on different timescales. Letting $T_{P}$, $T_{D}$, $T_{O}$ represent the timescales of protein dynamics, dye translational dynamics, and dipole orientational dynamics. The order of timescale separation assumed in this work is given by $T_{P}>>T_{D}\gg >T_{O}$. Further, as in [53], it is assumed that the orientation process and the translational process are independent processes. Note that this is an extremely common assumption since the independence of κ and r dynamics is implicitly assumed whenever the average κ value is used and whenever static κ distributions are employed. [68] Moreover, to provide a clear and succinct picture of the influence of dye dynamics on FRET measurements, the timescale $T_{P}$ is not considered in the current discussion. However, an extension of this analysis to include this timescale is in development.

### A. Spring Models

The simplest possible model to describe a stationary mean-reverting process is an Ornstein - Uhlenbeck (OU) process.[69] This physically represents an overdamped harmonic oscillator subject to noise.[70] The OU process is a Gauss-Markov process and, therefore, provides a simple model for thermal fluctuations of the fluorescent dyes. The equation of motion for the state vector $X_{t}\in R^{3}$ is given by the stochastic differential equation,

(12) $$dX_{t}=K\left(X_{t}-X_{eq}\right)dt+\sigma I\circ dB_{t},$$

where $K={\left[k_{i,j}\right]}_{i,j=1}^{3}$ is a matrix of spring constants. The notation $\circ dB_{t}$ denotes the use of Stratonovich integration.[71] We refer to systems such that the spring matrices can be written in the form $K=kI_{3\times 3}$, as isotropic springs. Otherwise, the system is called anisotropic.

Both isotropic and anisotropic spring systems with a diagonal spring matrix are considered. The spring coefficients are calculated using the linker chemistry. Utilizing the vibrational frequency of a C-C bond, we find that the spring constant for a single C-C bond is k=1010N/nm.[72] Therefore, a system of N≥1,C-C links is treated as a system of springs in series. Therefore

$$\frac{1}{k_{eff}}=\sum_{i=1}^{N}\frac{1}{k}\to k_{eff}=k\frac{k}{N}.$$

Finally, to find the length of the linker, we investigate the equilibrium bond length, L, in a C-C-C link. Using the law of cosines, we find that $2L=\sqrt{2l^{2}-2l^{2}cos\theta }$ with l being the length of a C-C bond. Therefore, the effective length in the linker for each link can be calculated using l=1.54Å and $\theta =109.5^{\circ }$.

**FIG. 4:**
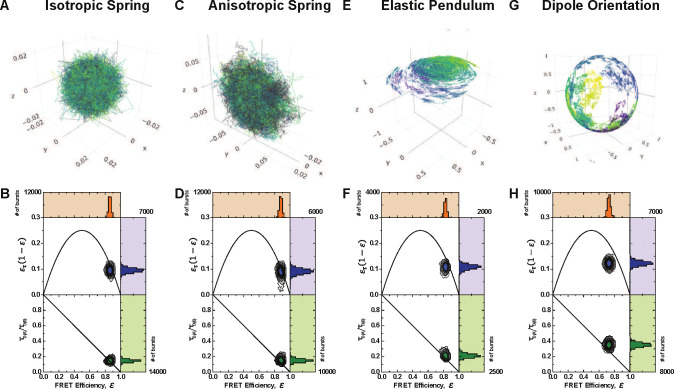
Dye model trajectories and resulting FRET efficiency vs normalized mean fluorescence lifetime and moments difference for A-B) Isotropic Spring, C-D) Anisotropic Spring, E-F) Elastic Pendulum. G) Sample trajectory of a spherical Brownian motion on the unit sphere. Such processes are used to model the diffusion of the electric dipole moment. H) FRET efficiency vs normalized mean fluorescence lifetime and moments difference when the dipole orientation for donor and acceptor is included in the FRET process during the Elastic Pendulum model.

In the isotropic case, illustrated in Figure 4A, the spring matrix is given by $k_{eff}I_{3\times 3}$. This provides a symmetric three-dimensional Gaussian as the stationary distribution for the isotropic spring.[69] It can be seen in [70, 73] that the variance of this distribution will be given by $\Sigma =\frac{\sigma }{k_{eff}}I_{3\times 3}$.

In the anisotropic case, illustrated in Figure 4C, we use a diagonal spring matrix with two entries being $pk_{\text{eff}}$ and the third being $k_{\text{eff}}$ with p∈(0,1). Therefore, the stationary distribution is an ellipse with major axes determined by the entries of the spring matrix.

This planar freedom in the anisotropic case can be used to investigate the influence of the orientation of the stationary distribution on the resulting dynamic shift. Such a scenario is exemplified in the case when the planes formed by the major axes of each stationary ellipse are mutually orthogonal. Since the stationary distribution for the isotropic case is a sphere and is perfectly symmetric, this can only arise in the anisotropic case.

Furthermore, these models have the added benefit of having an analytical expression for the inter-dye displacement, espeically in the isotriopic case. Sine the coordinates will be Gaussian distributed the distance between them is simply Reighley distributed. [64] This distribution is unimodal, and therefore the only mixing present is due to the variance of the stationary distributions. This mixing is, therefore, strongly dependent on the flexibility of the dyes, and as seen above, the variances in their positions when modeling the spring constant according to dye linker chemistry are quite small.

### B. Elastic Pendulum Model

The next model for the dye linker dynamics takes a stochastic geometric mechanics approach. Consider the motion of a rigid body attached to a spring that is free to move in space. This system forms an elastic pendulum.[74] The following system of Langevin equations describes the motion of a point mass elastic pendulum system subject to white noise;

(13) $$\left\{\begin{array}{ll} dr_{t} & =-k_{r}\left(r_{t}-r_{eq}\right)+\frac{1}{r_{t}}dt+\sigma_{r}\circ dB_{t}^{r}\\ d\theta_{t} & =-k_{\theta }sin\left(\theta_{t}\right)+\frac{\sigma_{\theta }^{2}}{r_{t}^{2}tan\;\theta_{t}}\circ dB_{t}^{\theta }\\ d\phi_{t} & =\frac{\sigma_{\phi }}{r_{t}sin\left(\theta_{t}\right)}\circ dB_{t}^{\phi }. \end{array}\right.$$

Importantly, the system is considered in spherical coordinates. The radial dynamics, $r_{t}$ evolve according to the spring dynamics explained in Section IIA, with slight alterations due to the change of coordinates. The angular parts of the motion are given by the standard nonlinear pendulum force in the polar direction $\theta_{t}$ and free diffusion in the azimuthal direction $\phi_{t}$. Figure 4 shows a sample dye trajectory.

The flexibility in the angular components is reminiscent of the wobble in a cone model used in previous investigations[44], and angular flexibility can be explained via the angular flexibility of C-C bonds themselves. However, unlike the classical wobble in a cone model, thermal noise and dye linker chemistry drive the dynamics and present a purely stochastic system. Moreover, by varying the parameters used, the system shows various behaviors.

Moreover, this model presents a possible explanation for the dynamic shift induced by dye motion due to the non-Gaussian inter-dye distributions, as shown in

This bimodality presents a mixing of two distinct states that are frequently needed to present a dynamic shift. Further, this provides a much larger change in inter-dye displacement than the spring models, which, as mentioned in Section IIA, do not possess any strong range of translational motion.

### C. Orientational Dynamics

The final consideration involves the orientational dynamics of the electric dipole moments of the dyes. As discussed in Section I, the Förster radius is dependent on the κ parameter, which is dependent on the mutual orientations of the electric dipole moments $\mu_{A}$, $\mu_{D}$ and the inter-dye displacement unit vector $\overset{ˆ}{R}$. Typically, $\kappa^{2}$ is taken as the mean value of 2/3 when the unit vectors are considered uniformly distributed on the sphere.[49, 50, 68] This assumption ignores the temporal aspect of the fluorescent process. Since dyes reorient on timescales faster than fluorescent lifetimes, the energy exchange rate changes during the FRET process. This changes the original CTMC model to a time-inhomogeneous CTMC, and thus, the transfer rates are dependent on the time integral of the infinitesimal transfer rates. [55–58]

To incorporate the influence of orientational dynamics on the lifetime distribution and FRET efficiency, consider the dipoles to be fixed to a reference frame of some rigid body with tensor of inertia I. The rigid body of the dye will be subjected to random torques and, therefore will reorient according to the Euler equations[74, 75]

(14) $$\left\{Id\omega_{t}+\omega_{t}\times I\omega_{t}=-\nu \omega_{t}+dW_{t}\;\omega_{t}=d\Phi_{t}\right.$$

where $dW_{t}$ is a spherical Brownian motion and $\Phi_{t}$ is the angular position vector. Assuming the dye is overdamped and hence $d\omega_{t}=0$, one obtains the simplified equations

(15) $$\left\{\begin{array}{l} \omega_{t}\times I\omega_{t}=-\nu \omega_{t}+dW_{t}\\ \omega_{t}=d\Phi_{t}. \end{array}\right.$$

Making the assumption that the dye is spherical and therefore the inertia tensor may be replaced with a scalar value [74] and using the fact that v×v=0 for any vector v we obtain the simple formula

(16) $$\nu d\Phi_{t}=dW_{t}$$

and hence, the dipole diffuses according to a spherical Brownian motion. Spherical Brownian motions can be expressed in terms of the Langevin equations below [76, 77]

(17) $$\left\{d\theta_{t}\;=\frac{\sigma_{\theta }^{2}}{tan\left(\theta_{t}\right)}dt+\sigma_{\theta }\circ dB_{t}\;d\phi_{t}\;=\frac{\sigma_{\phi }}{sin\theta_{t}}\circ dB_{t}\right.$$

The rotational diffusion coefficients depend on the hydrodynamic radius of the dye $R_{h}$ by the classical relation $D=8\pi \nu R_{h}^{3}$, where ν is the dynamic viscosity of the surrounding fluid. A sample trajectory is shown in Figure 3.

Note that the stationary distribution for such a system is the uniform distribution, providing an ideal starting stochastic process to test the time-dependent behavior of orientational dynamics.[71, 75] The key idea is that the excitation of the fluorophores provides a single initial κ value. This value then needs to evolve back to equilibrium during the FRET process. The relaxation effects are the object of interest, especially with regard to lifetime duration. The notion that κ may be close to 0 for the entire FRET process for one excitation but higher for another in the same sampling time provides an additional source of variance in the lifetime distribution. This should depend on the rotational diffusion of the dipole moment, with faster reorientation causing an averaging out effect as mentioned in [50].

## III. SOURCES OF OBSERVED DYNAMIC SHIFT

### A. Dye Configuration

This section compares the dye models mentioned in Section II. First, inspecting the joint FRET-lifetime distributions using the contour plots and marginal histograms seen in Figure 4, one can see the influence of the differing models. These FRET-lifetime distributions were generated by simulating the above-mentioned models in a confocal smFRET environment. As can be seen, the spring models show similar characteristics to the anisotropic model, which shows slightly more dynamic shifting. In addition, the elastic pendulum model produces a noticeable shift where the bulk of the distribution lies off of the static line. Despite the dynamic mixing involved in the purely translational elastic pendulum model, the dynamic shift produced is still not representative of experimental observations. Only through the addition of the orientational motion and time-inhomogenous energy transfer rates does the distribution show the hallmark dynamic shift in both the moment difference as well as direct FRET-lifetime distribution.

**FIG. 6:**
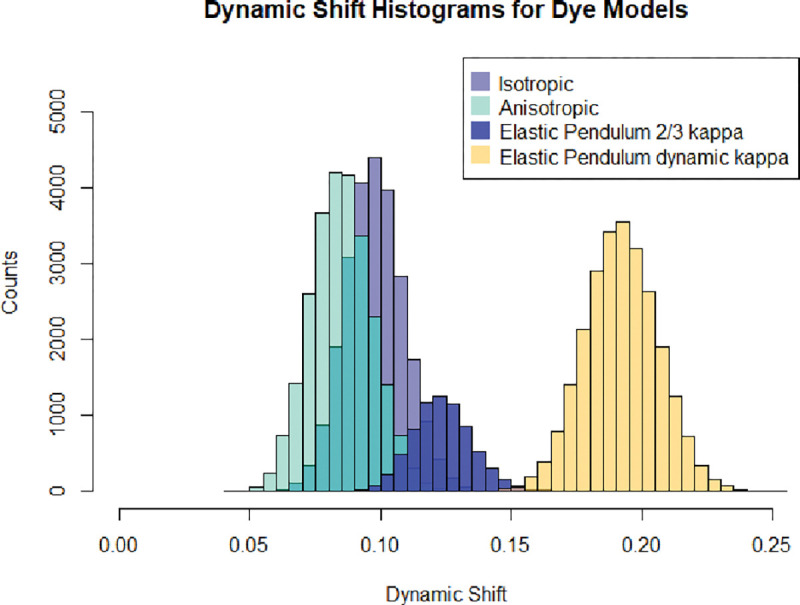
Linear Dynamic shift, d, histogram comparison of dye models

The dynamic shift distributions of each dye configuration are calculated using the definition of the dynamic shift shown in Equation (11). It has been known from experimental data that the average dynamic shift of dye motion is μ(Δ)≈0.2.[13] Using this quantity, the average dynamic shift for the associated models is examined to determine the model that captures the appropriate mean dynamic shift. The comparison of the dynamic shift distributions is shown in Figure 6.

In addition, these simulations have no burst noise from background radiation, as this could potentially cloud the impact of the dye motion.[62] The noise is solely from the experimental photon loss considerations and the dye motion as dictated by the models and simulation methods. Therefore, the only source of dynamic shifting must be from the dye models.

Curiously the dynamic shift densities shown in Figure 6 exhibit similar variances but differing mean dynamic shift values, as seen in Table I. While the elastic pendulum model does not completely capture the mean dynamic shift, with the addition of a dynamic $\kappa^{2}$ value, the proper dynamic shift appears. This is in stark contrast to previous suggestions that the dynamic shift is a result of the accessible volume of the dye. Despite the elastic pendulum having an accessible volume comparable to those found in all-atom molecular dynamic simulations and exhibiting dynamic mixing between two states, the resulting dynamic shift is less than expected. This suggests that the inclusion of time-inhomogeneities in the Förster radius due to orientational factors is essential to the description of the dynamic shift.

It is important to note that the spring models can be made to incorporate a dynamic $\kappa^{2}$ parameter. However, the model parameters based on linker composition prevent these models from being used in earnest, as the accessible volumes are smaller than physically reasonable. This can be seen by investigating the variance of their associated stationary distributions. As mentioned in Section IIA, the variance along each axis in the isotropic case will be $\sigma /k_{\mathrm{eff}}$, or $K_{B}T/\gamma k_{\mathrm{eff}}$, where γ is the local friction. This produces a stationary distribution with a 3 standard deviation radius of less than an Angstrom.

### B. $\kappa^{2}$ Dynamics

**FIG. 7:**
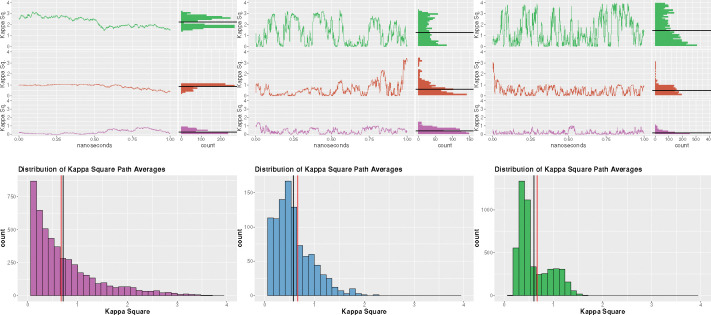
Comparison of $\kappa^{2}$ trajectories for three common cases. Figure *A* − *C* shows the sample paths of $\kappa^{2}$ during an excitation event, these can be seen as four realizations of $\kappa^{2}$ during the same burst. Each path is representative of a single energy transfer event. Figures *D* − *F* show the associated distribution of average $\kappa^{2}$, the red vertical lines in figures *D* − *F* are the isotropic 2/3 average, where the black lines are the mean of the path average. The black lines in Figures *A* − *C*, for all excitation events in a burst. Figures *A* and *D* show a pair of dyes for which the rotational diffusion is only an order of magnitude greater than the translational diffusion of the dye. Figures *B* and *E* show the sample behavior when one dye has a rotational diffusion three orders of magnitude greater than translational and one dye one order of magnitude greater. Finally, Figures *C* and *F* show the case in which both dyes have rotational diffusions three orders of magnitude greater than translational.

A chief consideration brought to attention in Section IIIA is the influence of $\kappa^{2}$ dynamics on FRET lifetime pairs. The key issue in incorporating $\kappa^{2}$ dynamics into FRET uncertainty quantification has been to assess $\kappa^{2}$ as a stationary object during energy transfer. This is done by considering $\kappa^{2}$ as being chosen from its equilibrium distribution, assuming that it follows a discrete state Markov chain, or simply using the 2/3 approximation. [53, 78–80] From there, one can use the mean and standard deviation in uncertainty quantification.[49, 50, 68] However, these approximations ignore the time inhomogeneous nature of the FRET process, as mentioned in Section IB. Notably, the probability of a donor fluorescence event, as shown in Section IB, is dependent on the integral of the energy transfer rates as well as the lifetime distribution. [55, 56, 58, 81] While the use of an ergodic approximation might mitigate these concerns, this relies on the convergence of the long run time average to the long run spatial average. The short time span involved in the FRET process, especially in slow rotational diffusion regimes, should not be considered long enough to invoke such an approximation. Furthermore, this mitigation ignores the dependence of the energy transfer rate and FRET efficiency on the history of the $\kappa^{2}$ process. As seen in Section IB, the energy transfer rate is dependent on the integral of the $\kappa^{2}$ path. For example, in Figures 7A and B, the second (red) trajectories have similar mean values after the 1*ns* run, with path A having the larger. However, if one were to investigate the energy transfer rate at time 0.5 one would find that the probability of energy transfer is much lower for path B rather than being similar to path A. One can see how this integral fluctuation may be reduced in the presence of fast dyes. As shown in Figure 7C, the rapid oscillations induced by the swift rotational diffusion of the dyes produce highly clustered $\kappa^{2}$ trajectories. However, as can be seen in the associated average $\kappa^{2}$ distribution in Figure 7F, the resulting average distribution becomes bimodal. This bimodality can be seen in the trajectories in which the oscillations can vary between smaller values or larger values. Notably, this is heavily dependent on the radial-dipole dot product term in the definition of $\kappa^{2}$. Furthermore, with the decrease in rotational diffusion, the $\kappa^{2}$ process predictably oscillates much slower. This yields trajectories such that larger values are maintained for longer. These larger stretches of high $\kappa^{2}$ values can lead to much shorter donor lifetimes, but similarly, this also leads to longer stretches of small $\kappa^{2}$ values. Overall, slower rotational diffusion will show a larger temporal correlation. Hence, the stationary distribution will play a much more important role, since the $\kappa^{2}$ trajectory is not as likely to change as drastically from its initial value.

The skew of the mean $\kappa^{2}$ value shows the flaw in the averaging assumption. While the mean $\kappa^{2}$ average is indeed 2/3, the most common values for the mean $\kappa^{2}$ are below this value. In fact, one can see that out of the three distributions of average $\kappa^{2}$ the only one in which the most common value is 2/3 is Figure 7E, wherein as mentioned above, the temporal fluctuations of the $\kappa^{2}$ path will play a larger role due to their larger time inhomogeneity than the paths in Figure 7A and 7C.

The dynamic shift produced by dye motion is then a product of the variability of the $\kappa^{2}$ trajectories. This temporal inhomogeneity *during* the energy exchange process provides the crucial mixing element to significantly slow the the average sampled donor lifetime. While it can be seen in Figure 4 that inter-dye radial displacement dynamics can produce a slight dynamic shift, the mixing is not strong enough to produce the lifting seen by considering the direct change in energy transfer rate caused by rotational dynamics. Figure 7D–7E can be seen as representative of the average $\kappa^{2}$ values during a single burst. If one simplifies the path-based description above to these distributions, it is simple to see that in the case of two rapidly rotating dyes, the bimodality of the distribution provides two populations of energy transfer rate. The switching between the two during the burst provides a direct comparison to the two-state systems investigated in [13, 14]. However, it must be stressed that the path-by-path inhomogeneity of the FRET process is crucial to the analysis. While the average values shown can be representative of normalized time integrals, one must consider that the plots shown in Figure 7 each last for one nanosecond, and therefore do not capture the stopping time of fluorescence or energy transfer. As noted, the time-dependent FRET efficiency ℰ(t) will vary path-by-path, producing the observed lifetime distribution.

## IV. DISCUSSION

From the above results, it has been shown that the dynamic shift resulting from dye motion is not as simple as previously thought. By presenting the first physics-based model for fluorescence dynamics, incorporating dye linker chemistry and fluorescent dye composition, it has become clear that the time-inhomogeneous nature of the Förster radius is an essential part of the dynamic shift. This is shown by noting that models with proper accessible volumes do not demonstrate experimentally observed dynamic shifts and noting that the time-inhomogeneous nature of the FRET process depends on the path space dynamics of $\kappa^{2}$ trajectories. Furthermore, it was shown that the dynamics of $\kappa^{2}$ trajectories change with the selection of fluorescent dyes used, with common situations such as an organic dye paired with a fluorescent protein exhibiting noticeable differences from a pair of organic dyes. While the average $\kappa^{2}$ stays consistent with the 2/3 isotropic assumption, the fluctuations during the FRET process cause fluctuations in the FRET-lifetime distribution during the burst. These fluctuations provide a source of dynamic mixing and, hence, a dynamic shift.

Additional considerations of dye orientational dynamics are not here considered due to the added complexity. In this work the results are for only spherical dyes, however, the rotational Langevin equations used in section IIC can be utilized for arbitrary intertia tensors. While this is simple to state, the resulting equations become arbitrarily difficult to work with, as well as simulate. Such an endeavor is beyond the scope of the present work, but is highly encouraged as a future research direction since different intertia tensors will produce different rotational processes and hence different $\kappa^{2}$ trajectories. Further considerations into the coupling between translational motion and orientational motion must also be considered. Unfortunately, the incorporation of this consideration is distracting to the message presented above and comes with unique challenges. While this is a common assumption, as it is invoked each time dynamic averaging is used for $\kappa^{2}$ and is used in other theoretical analyses such as [53] it is an important direction of consideration. As noted in [68] there is a marked correlation between translational motion and $\kappa^{2}$. This can be further emphasized by the considerations of Sections IIB and IIC wherein one may couple the orientational rigid body dynamics and the dynamics of the elastic pendulum. Such a system is known to have non-trivial coupling in the classical case that is likely to remain in the overdamped case. These considerations, however, increase the difficulty of analysis and simulation considerably as one must consider the dynamics of a full state space model on the non-compact, non-abelian Euclidean group SE(3) [75] rather than independent processes as used here and in [53].

Furthermore, the work shown here provides an important framework for analyzing other sources of the dynamic shift. The algorithms used to simulate the experiments can easily be scaled to incorporate another timescale. This opens the door to studying the influence of protein dynamics on the dynamic shift. By using reaction coordinate Langevin models, one may investigate the influence of differing energy landscapes on the dynamic shift, as well as the influence of a more dynamic inter-dye distance vector. This can be done swiftly due to the low computational cost incurred by the stochastic simulation of the FRET process. Additionally, by introducing a new form for the dynamic shift, distributional quantities may be accessed more readily, providing a new tool to analyze the motion of underlying molecules of interest as well as dye dynamics. Additionally, while our simulated experiments were conducted in the confocal environment the results carry over to TIRF measurements as well.

In conclusion, this work has demonstrated the importance of time-inhomogeneities in the FRET process and their influence on resulting measurements. The path dependence of the FRET efficiency, as well as the influence of a time-varying Förster radius during the FRET process, is shown to be non-negligible. In order to conduct optimal uncertainty quantification for smFRET measurements the path dynamics of $\kappa^{2}$, not just the average values, must first be understood. Therefore, the anisotropic behaviors of fluorescent dyes are of vital importance. By gaining an understanding of the orientational dynamics of fluorescent dyes, better uncertainty quantification for FRET measurements can be done. Additionally, this relationship between the full state space dynamics of a fluorescent dye and resulting smFRET measurements emphasizes the need for more stochastic geometric mechanical considerations in fluorescent measurements and biology.

## Figures and Tables

**FIG. 5:**
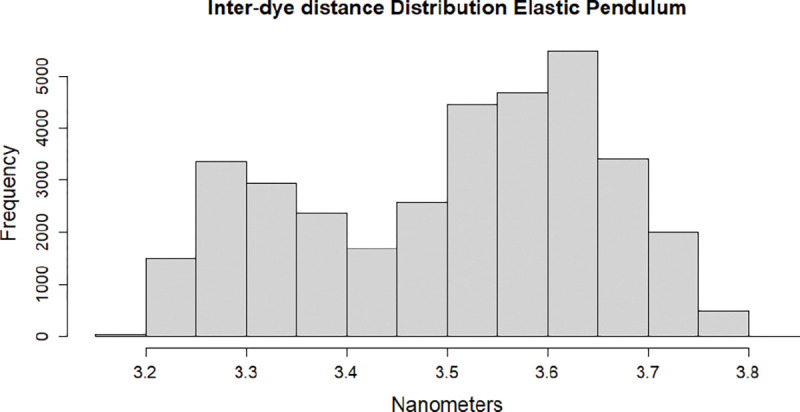
Histogram of inter-dye distances for the elastic pendulum model during an excitation event.

**TABLE I:** Dynamic Shift: Mean and standard deviation of the dynamic shift for each model in Figure 6.

Model	µ(∆)	σ(∆)
Isotropic Spring	0.10	0.01
Anisotropic Spring	0.01	0.01
Elastic Pendulum κ≈2/3	0.12	0.01
Elastic Pendulum Dynamic κ	0.20	0.10
